# Carcinogenic Effects in a Phenylketonuria Mouse Model

**DOI:** 10.1371/journal.pone.0004292

**Published:** 2009-01-27

**Authors:** Neil Sidell, Lijuan Hao, Marzia Pasquali, J. David McDonald

**Affiliations:** 1 Department of Gynecology and Obstetrics, Emory University School of Medicine, Atlanta, Georgia, United States of America; 2 Department of Pathology, University of Utah, Salt Lake City, Utah, United States of America; 3 Department of Biological Sciences, Wichita State University, Wichita, Kansas, United States of America; Universidad Nacional Mayor de San Marcos, Peru

## Abstract

Phenylketonuria (PKU) is a metabolic disorder caused by impaired phenylalanine hydroxylase (PAH). This condition results in hyperphenylalaninemia and elevated levels of abnormal phenylalanine metabolites, among which is phenylacetic acid/phenylacetate (PA). In recent years, PA and its analogs were found to have anticancer activity against a variety of malignancies suggesting the possibility that PKU may offer protection against cancer through chronically elevated levels of PA. We tested this hypothesis in a genetic mouse model of PKU (PAH^enu2^) which has a biochemical profile that closely resembles that of human PKU. Plasma levels of phenylalanine in homozygous (HMZ) PAH^enu2^ mice were >12-fold those of heterozygous (HTZ) littermates while tyrosine levels were reduced. Phenylketones, including PA, were also markedly elevated to the range seen in the human disease. Mice were subjected to 7,12 dimethylbenz[a]anthracene (DMBA) carcinogenesis, a model which is sensitive to the anticancer effects of the PA derivative 4-chlorophenylacetate (4-CPA). Tumor induction by DMBA was not significantly different between the HTZ and HMZ mice, either in total tumor development or in the type of cancers that arose. HMZ mice were then treated with 4-CPA as positive controls for the anticancer effects of PA and to evaluate its possible effects on phenylalanine metabolism in PKU mice. 4-CPA had no effect on the plasma concentrations of phenylalanine, phenylketones, or tyrosine. Surprisingly, the HMZ mice treated with 4-CPA developed an unexplained neuromuscular syndrome which precluded its use in these animals as an anticancer agent. Together, these studies support the use of PAH^enu2^ mice as a model for studying human PKU. Chronically elevated levels of PA in the PAH^enu2^ mice were not protective against cancer.

## Introduction

About the time of the discovery of phenylketonuria (PKU) in 1934 [1], it was reported that patients with this disease excreted abnormally high levels of phenylalanine metabolites in their urine. These metabolites included a variety of compounds that normally arise when phenylalanine is not broken down in the normal fashion by the liver enzyme phenylalanine hydroxylase (PAH), resulting in an alternate pathway of phenylalanine metabolism. These observations led to the discovery of the causative factor of PKU as being a lack of PAH activity due to one or more mutations in the gene coding for this enzyme [2]. One of the compounds that was shown to be markedly elevated in the urine of PKU subjects was the phenylalanine metabolite phenylacetic acid [3]. This compound, or the ionized form of the molecule, phenylacetate (PA), is usually undetectable in plasma of normal individuals [4]. PA has been used for a long time in the treatment of conditions associated with hyperammonemia (e.g. urea cycle disorders) [5], [6]. This experience has indicated that millimolar blood serum levels of PA can be achieved without significant adverse effects; prolonged treatment with PA (up to 6 years) of children with inborn errors of urea synthesis have not shown significant signs of toxicity [7]. This lack of toxicity, along with studies demonstrating the anticancer properties of PA derivatives against a variety of malignancies, prompted intense investigation of these compounds in anticancer applications [8]–[11]. To this end, our previous work demonstrated that PA and its halide derivative 4-chlorophenylacetate (4-CPA), can retard the growth of estrogen-dependent breast cancer cells in vitro, and the development of breast cancer in an in vivo mouse model [11], [12]. Utilizing transgenic mice which exhibit chronically elevated levels of estrogen in their mammary glands, these latter studies showed that 4-CPA inhibited the development and growth of estrogen-driven breast tumors. This antiestrogenic activity may be due to the ability of PA and 4-CPA to interfere with estrogen signaling through their direct binding to estrogen response element regulatory sites in the promoters of target genes [13].

This information inexorably leads one to ask the following question. Since PA is one of the metabolites of phenylalanine that is elevated in individuals with PKU, is the cancer incidence/types/cancer mortality different in this population of people? If so, PKU may be “nature's model” for assessing the anticancer properties of this compound. To our knowledge, no such study has yet been conducted. One reason for the dearth of information on this subject may be that the oldest early-detected adults with PKU are only now in their 40′s, since national newborn screening did not start until 1963 [14]. With a population that young, and the cancer incidence among such a young group very low, coupled with the small population size to begin with (total PKU population of all ages of early-treated persons is probably less than 15,000 nationally), it may be difficult to discover anything definitive about the protective effects of phenylacetate among these individuals. A more interesting population to look at might be the older untreated adults with PKU, where one would expect more cancer due to age. In addition, given their untreated PKU, this population would likely have the highest amounts of phenylacetate in their blood. However, for the most part these untreated individuals are institutionalized, and tracking down significant number would be difficult. Thus, although there are some states (e.g. California and Minnesota) which maintain PKU registries to facilitate acquisition of this data, it is clear that, at this time, obtaining a definitive answer to these questions may not be possible. As a result, we have tested our hypothesis that PKU may protect against certain kinds of cancers by utilizing a well-characterized PKU mouse model and a 7,12-dimethylbenzanthracene (DMBA)-induced tumor induction protocol. Our results did not show a significant difference between the development of tumors in PKU and control mice, and therefore do not support the above hypothesis.

## Results and Discussion

### Phenylalanine, phenyketone, and CYP1B1 levels

Table 1 shows that phenylalanine and phenylketone levels, known to be elevated in human PKU, were elevated in the PKU homozygous PAH^eun2/eun2^ (HMZ) mice and in the range reported in the human disease [4], [15]. Tyrosine was reduced in the HMZ mice, also mimicking human PKU. Levels of phenylalanine and tyrosine in the heterozygous PAH^enu2/wild^ mice (HTZ) were similar to that seen in the wild-type BTBR strain and in normal humans [15], [16]. To our knowledge, plasma levels of these PKU-related phenylketones in the HMZ mice have heretofore not been reported. These analyses provide striking new evidence of the remarkable similarities between murine PKU as represented in this PAH^enu2^ animal model and human PKU. Together with reported genetic, neurological, and biochemical comparisons with human PKU [16]–[18], our findings emphasize the physiologic relevance of this mouse model for studying the human disease.

**Table 1:** Amino Acid and Phenylketone Levels in Plasma of PKU Mice^a^,^b^

Genotype	Phenylacetate	Phenyllactate	Phenylpyruvate	Tyrosine	Phenylalanine
Heterozygous	n.d.^c^	n.d.	n.d.	86.3±10.1	102.7±14.8
Homozygous	12.5±1.7	16.1±2.5	51.0±8.1	35.2±5.2	1405.2±115.9

a All values are in μM/L and represent mean SEM of six mice in each group.

b Determined by GC/MS as reported in our earlier work (34).

c not detected

DMBA is a “pro-carcinogen” and must be metabolized to produce the proximate carcinogenic metabolite 3,4-dihydrodiol [19]. The main enzyme responsible for the catalytic conversion of DMBA to an active carcinogen is the P450 enzyme CYP1B1 [19]. This conversion takes place mainly in the liver. CYP1A1, although also contributing to the metabolism of DMBA, is thought to only play a minor role in this process. Thus, potential differences in tumor induction between HMZ and control animals could reflect differences in the levels of these enzymes which would correspondingly affect the carcinogenic potential of administered DMBA. To address this potential complication in our study, we measured the activity of these enzymes using ethoxyresorufin-O-deethylase (EROD) as a surrogate substrate for DMBA [12]. This compound is specifically catalyzed by CYP1A1 and CYP1B1 into a fluorescent metabolite that can easily be measured by spectrophotometry. As expected from previous mouse studies [12], [19], no activity was detected in mammary tissue from either the control or HMZ mice (n = 3). Both groups showed activity in liver as follows: HTZ = 7.0±2.0; HMZ = 10.6±1.0 pmol resorufin/min/per mg protein (p = 0.18, not significant). Thus, there was no significant difference between HTZ and HMZ mice in the conversion of DMBA to its carcinogenic metabolite that might otherwise have complicated the interpretation of possible differences in tumor induction between the groups.

### Tumor development

**Figure 1:**
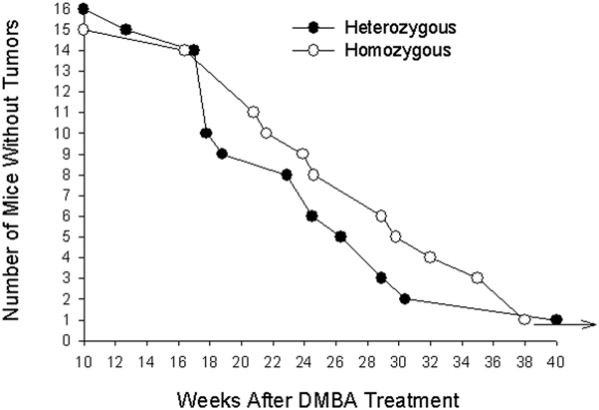
Tumor development. Female HTZ and HMZ PAH^enu2^ mice as indicated were subcutaneously implanted with a slow releasing MPA pellet at approximately 5 weeks of age, then treated with DMBA p.o. (1 mg/mouse) approximately 2 weeks later. Mice were given a total of 4 treatments of DMBA (one/week for 4 weeks). Tumor development in mice was assessed as described in Materials and Methods.

**Figure 2:**
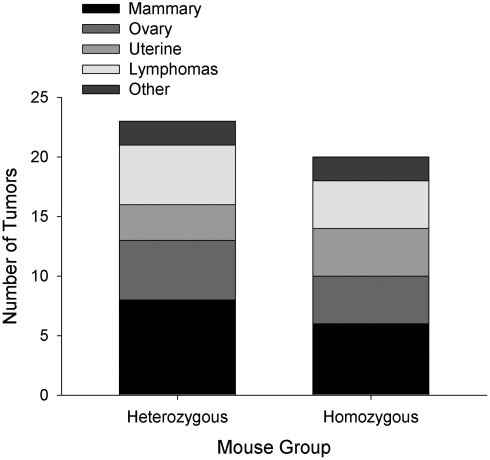
Tumor types induced in the HTZ and HMZ mice.

To determine whether the HMZ mice show a reduced incidence of tumor induction, we employed a protocol similar to that previously used in our aromatase transgenic mouse study [12], with the exception that slow release (90 day) MPA pellets were added to the treatment to increase the incidence of tumors induced [20]. MPA treatment was not necessary in the transgenic mouse study due to the chronically elevated estrogen levels in the mammary tissue of these mice [21]. For these experiments, 4 mice in the HTZ group and 5 in the HMZ group expired before the experiment was terminated and no tumors were discovered upon necropsy. Therefore, these mice were not counted. As seen in figure 1, tumors started to appear in both the HTZ and HMZ groups between 12 and 16 weeks after the last dose of DMBA and there were no significant differences in subsequent tumor development between the groups at any time point (two-tailed Fisher's exact test). The mean tumor latency (±s.d.) for the HTZ and HMZ mice was 22.9 (7.4) and 27.1 (6.9) weeks, respectively. Using the log-rank test, the development of tumors between the two groups is not significant (p-value of 0.37) which is consistent with the results from the Fisher's exact test. In contrast to our previous study in which the overwhelming majority of tumors that were induced in DMBA-treated aromatase transgenic mice were breast cancers [12], a variety of tumor types developed in both the HTZ and HMZ mice including ovarian, uterine, mammary cancers, and lymphomas. These tumor types have previously been reported to be induced by the DMBA/MPA treatment protocol used in this study [22], [23]. As shown in figure 2, there was no significant difference in the distribution of these tumor types between the two groups. However, it should be noted that the number of mice used in these experiments was not sufficient for detecting differences in the development of the specific tumor types found in the study groups. Indeed, based on the tumors showing the largest prevalence as well as the greatest difference in occurrence between the HMZ and HTZ groups (mammary tumors), at least 220 mice in each group would be required to detect significant differences relating to the development of specific tumor types (two-tailed Fisher's exact test). We did not feel that such an expanded study was warranted based on the present findings.

These results do not support the hypothesis that chronically elevated PA at serum levels seen in the HMZ mice (∼13 μM) can influence DMBA-mediated tumor induction. As such, these results did not provide evidence that PKU can protect against cancer. A possible reason for the lack of PA effects in this study versus the potent suppression of 4-CPA on tumor induction in aromatase-transgenic mice could be that tumors induced in the latter are almost exclusively estrogen-driven [12], [21]. In contrast, although we did not determine the ER status of tumors arising in the present work, similar induction protocols in a variety of mouse strains have been shown to produce predominantly ER-negative (i.e. estrogen independent) tumors [22]–[25]. Alternatively, both 4-CPA and PA have been shown to be relatively weak antiestrogens and concentrations of the compounds needed to effectively block the action of ER in vitro were found to be in the mM range [26]. This concentration range was achieved in the aromatase-transgenic mouse experiments [12] while levels of PA detected in the HMZ mice were only in the μM range.

When interpreting these results, we emphasize that effects of certain carcinogens have been shown to be dependent on age, sex, and animal strain in different mouse models. For example, Horlings and Demant [27] demonstrated strain-specific differences in lung tumor induction by ethylnitrosourea ENU while variations between male and female mice in responses to protocols involving ENU in promoting hepatocarcinogenesis have also been documented [28]. Carcinogenic studies of the parental strain of our HMZ or HTZ mice (BTBR) have, to our knowledge, not been reported, including that by ENU which was used in the creation of this PKU model [17]. Thus, care should be taken not to “over generalize” the results obtained here to PKU conditions of varied genetic backgrounds.

### Treatment of mice with 4-CPA

In PKU, there is defective hydroxylation of phenylalanine to produce tyrosine. This condition results in reduced levels of tyrosine metabolites such as thyroxin, melanin, and the dopamine family of neurotransmitters [29], [30]. The reduced levels of these products can cause a variety of abnormalities associated with PKU such as hypopigmentation and derangement of the dopaminergic system although impaired cognitive function seems directly related to the elevated phenylalanine levels. Physiologically, tyrosine is converted to parahydroxyphenylpyruvate which is then further converted by the enzyme dioxygenase to homogentisic acid [29]. Interestingly, one of the phenylketone products that is elevated in PKU, phenylpyruvate (PPY), is structurally related to parahydroxyphenylpyruvate, the dioxygenase substrate for homogentisate synthesis, and can act as a competitive inhibitor of this reaction [31], [32]. Thus, concentrations of PPY similar to that found in plasma of some treated PKU patients have been shown to impair tyrosine degradation through this pathway [33]. Since PA is a metabolite of PPY, we tested the possibility that an increase in plasma levels of 4-CPA in the HMZ mice could alter PPY levels such as to inhibit tyrosine degradation and increase its plasma concentration. In addition, treatment of mice with 4-CPA would also serve as a positive control in order to substantiate that the numerous metabolic abnormalities in the HMZ mice [16]–[18] do not antagonize the anticancer properties of PA compounds. In our previous work, we showed that long-term administration of 4-CPA in the drinking water of an aromatase-transgenic mouse model of breast cancer induction effectively inhibited tumor development [12]. These studies also demonstrated that this treatment for many months had no apparent adverse effects on the mice; there were no significant differences between 4-CPA-consuming and control groups in their water consumption, growth rate, social behavior, or vitality.

Table 2 shows that while 4-CPA was not detected in the plasma of untreated HTZ or HMZ mice, comparable levels of the compound (∼0.5 mM) were achieved in both groups following 2 weeks treatment by admixing 4-CPA in their drinking water. This plasma concentration of 4-CPA was similar to that achieved in our previous work [12], [34]. Table 2 also shows that administration of 4-CPA did not significantly affect the levels of phenylketones, tryrosine, or phenylalanine in the plasma of the HMZ or HTZ mice. Therefore, administration of PA derivatives did not alter the metabolic degradation of tyrosine such as to result in its increased plasma levels. However, in contrast to the complete lack of side effects of 4-CPA seen in our previous study and in the HTZ mice in the present work, the HMZ mice developed an unexplained neuromuscular syndrome which became apparent after the first week of 4-CPA treatment. This syndrome was characterized by limb weakness, hypermetria, and ataxia, and was progressive; by two weeks, weight lose of the mice was evident which appeared to be due to difficulty in walking and feeding. These adverse effects were only observed in the HMZ mice and prevented our testing of the anticancer properties of 4-CPA in these animals. Necropsy of the mice and histological analysis of the limb musculature did not reveal any lesions or apparent abnormalities. This interesting phenomenon was reversible; when the 4-CPA-containing water was replaced by normal water, all symptoms dissappeared within a few days. The characterization of this interesting syndrome and the determination of its cause will be the goal of future studies.

**Table 2:** Amino Acid and Phenylketone Levels in Plasma of Treated Mice^a^,^b^

Genotype	Treatment	Phenylacetate	Phenyllactate	Phenylpyruvate	4-CPA^c^	Tyrosine	Phenylalanine
Heterozygous	Untreated	n.d.^d^	n.d.	n.d.	n.d.	98.0±9.1	111.3±23.5
	4-CPA^e^	n.d.	n.d.	n.d.	517.5±38.0	107.7±8.8	107.3±9.4
Homozygous	Untreated	12.0±1.4	12.3±2.6	44.9±7.5	n.d.	39.0±9.0	1453.7±5.2
	4-CPA	18.1±0.5	15.8±2.4	36.2±2.2	428.6±62.1	27.0±6.3	1051.3±77.9

a All values are in μM/L and represent mean±SEM of three mice in each group.

b Determined by GC/MS as reported in our earlier work (34).

c 4-chlorophenylacetate

d not detected

e 4-CPA added to drinking water for 2 weeks at a concentration of 6 ng/ml.

## Materials and Methods

### Maintenance and treatment of mice

All procedures were carried out in accordance with the National Institutes of Health Guidelines on the Care and Use of Animals and an animal study protocol approved by Emory University's Institutional Animal Care and Use Committee. Mice used in this study were created in the BTBR strain by ENU germline mutagenesis in the gene for phenylalanine hydroxylase (PAH) which results in a loss of enzyme activity such that, in liver, phenylalanine hydroxylase activity is minimal [16]. The homozygous mutant BTBR-PAH^enu2^ mouse has shown to be a remarkably good model for human PKU. These animals show no residual activity of the liver PAH along with very high plasma Phe levels. Heterozygous PAH^enu2/wild^ (HTZ) and homozygous PAH^enu2/enu2^ (HMZ) mice were produced by crossing HMZ fathers and HTZ mothers. In our experiments, only female mice were utilized with the HMZ experimental group and were compared with female HTZ animals as the control group. Due to the well known carcinogenic effects of ENU [27], [28], we felt HTZ rather than wild-type BTBR mice to be a better control group in order to best isolate differences in tumor induction specifically due to the PKU phenotype. Mice who are heterozygous for PAH^eun2/wild^, like humans who are heterozygous for the PKU genotype, have plasma Phe levels that are similar to wild-type who carry no PAH mutation [16]–[18]. Genotyping for the homozygous versus heterozygous animals was not necessary since the homozygous mice were easily discernable by their lightened (grayish) coat color due to reduced melanin production.

To get reproducible tumor induction in the mice, we used a combination of oral administration of 7,12-dimethylbenz[a]anthracene (DMBA) along with subcutaneous implantation of a slow releasing (90-day) medroxyprogesterone acetate (MPA) pellet [20]. Briefly, Female HTZ or HMZ mice were subcutaneously implanted with the MPA pellet at approximately 5 weeks of age, then started DMBA treatment 2 weeks later. The DMBA (1.0 mg) dissolved in 100 μl of corn oil was delivered via orogastric tube to mildly anesthetized mice once per week for 4 weeks. One month after the administration of DMBA, the mice were palpated for tumors and weekly observations were continued up until 10 months after DMBA treatment. Mice were sacrificed shortly after tumor identification, if noticeably ill, or at the termination of the experiment. In selected mice, blood was obtained by cardiac puncture into heparinized syringes and, after centrifugation, the plasma was stored at −80 C until analysis by gas chromatography/mass spectroscopy (GC/MS)[34].

Treatment of mice with 4-CPA was performed by continuous intake of the compound in the animal's drinking water at a concentraion of 6 mg/ml as previously reported by our group [12]. We have shown that this method of long-term 4-CPA administration for many months has no adverse effects on the mice and achieves a mean 4-CPA plasma concentration of ∼0.8 mM.

### Liver P450 enzyme activity and plasma protein analysis

Phenylalanine and metabolites in the plasma of HTZ and HMZ mice were analyzed by an amino acid analyzer and GC/MS as described [34]. Quantitation of 4-CPA was performed by gas chromatography/mass spectroscopy (GC/MS) following ethylacetate/ether extraction of plasma deproteinized with 7% perchloric acid, according to standard procedures. Specifically, 250 μL of plasma was admixed with 250 μL of 7% perchloric acid. The mixture was centrifuged and the supernatant used for the analysis. The quantitation was accomplished by Selective Ion Monitoring (SIM) using an external calibration curve obtained with a standard undergoing the same extraction procedure as the samples. Corrections for variations in the injections were made with an internal standard (C24). The response was linear over a wide range of concentrations (0–1700 nmoles).

To measure CYP1B1 activity, the ethoxyresorufin-O-deethylase (EROD) assay was used [12], [35]. Reactions were performed in a total volume of 250 μl in microfuge tubes at 37°C at a final substrate concentration of 4 μM. Microsomal protein (100–250 μg) in storage buffer (10 mM Tris OAc, pH 7.4, 0.1 mM EDTA, 23% glycerol) was added to 7-ethoxyresorufin dissolved in DMSO and then diluted in double distilled water (final DMSO concentration of 0.08% v/v). The reaction mixture was allowed to equilibrate for 5 min at 37°C. Reactions were initiated by timed additions of 50 μL NADPH (10 mM). After 10 min incubation at 37°C, the reaction was stopped by timed additions of 250 μL ml of ice-cold acetronitrile. EROD activity was determined by measuring the fluorescence of resorufin at 545 nm (excitation) and 610 nm (emission) in a microplate reader. Resorufin standard curves were used for the determination of the activities.

### Assessment of tumors in mice

After the mice were sacrificed, necropsy and tumor evaluation was performed by the Diagnostic Laboratory, Division of Animal Resources, Emory University. The following tissue were collected and examined for gross lesions or abnormalities: heart, lungs, mammary glands, reproductive organs, liver, spleen, kidneys, stomach, cecum, large and small intestine, brain, salivary glands, thymus. Any suspicious tissue, tumors, and routinely mammary glands (as being the main target of DMBA-induced tumors) were dissected free from skin and processed for histology as previously described [12]. Routine sections of tissues were prepared after fixation in 10% neutral buffered formalin by embedding in paraffin, sectioning at 5 μm, and staining with H&E. Tumors were identified as described previously [12].

## References

[1] Fölling A (1934). Über Ausscheidung von Phenylbrenztraubensäure in den Harn als Stoffwechselanomalie in Verbindung mit Imbezillitäts.. Hoppe-Seylers Z Physiol Chem.

[2] Guldberg P, Henriksen KF, Guttler F (1993). Molecular analysis of phenylketonuria in Denmark: 99% of the mutatiions detected by denaturing gradient gel electrophoresis.. Genomics.

[3] Michals K, Lopus M, Matalon R (1988). Phenylalanine metabolites as indicators of dietary compliance in children with phenylketonuria.. Biochem Med Metab Biol.

[4] Tuchman M, Fisch RO, Ramnaraine ML, Krivit W (1985). Acidic metabolites of phenylalanine in plasma of phenylketonurics.. Biochem Med.

[5] Brusilow SW, Danney M, Waber LJ, Batshaw M, Burton B (1984). Treatment of episodic hyperammonemia in children with inborn errors of urea synthesis.. N Engl J Med.

[6] Scaglia F, Carter S, O'Brien WE, Lee B (2004). Effect of alternative pathway therapy on branched chain amino acid metabolism in urea cycle disorder patients.. Mol Genet Metab.

[7] Batshaw ML, Monahan PS (1987). Treatment of urea cycle disorders. Recent Adv Inborn Errors of Metabolism.. Enzyme.

[8] Samid D, Shack S, Sherman LT (1992). Phenylacetate: a novel nontoxic inducer of tumor cell differentiation.. Cancer Res.

[9] Hudgins WR, Shack S, Myers CE, Samid D (1995). Cytostatic activity of phenylacetate and derivatives against tumor cells: Correlation with lipophilicity and inhibition of protein prenylation.. Biochem Pharmacol.

[10] Sidell N, Wada R, Han G, Chang B, Shack S (1995). Phenylacetate synergizes with retinoic acid in inducing the differentiation of human neuroblastoma cells.. Int J Cancer.

[11] Sawatsri S, Samid D, Malkapuram S, Sidell N (2001). Inhibition of estrogen-dependent breast cell responses with phenylacetate. Int J Cancer.. 2001.

[12] Sidell N, Kirma N, Morgan ET, Nair H, Tekmal RR (2007). Inhibition of estrogen-induced mammary tumor formation in MMTV-aromatase transgenic mice by 4-chlorophenylacetate.. Cancer Lett.

[13] Sidell N, Tanmahasamut P, Ewing DE, Hendry LB (2005). Transcriptional inhibition of the estrogen response element by antiestrogenic piperidinediones correlates with intercalation into DNA measured by energy calculations.. J Steroid Biochem Mol Biol.

[14] Guthrie R, Susi A (1963). A simple phenylalanine method for detecting phenylketonuria in large populations of newborn infants.. Pediatrics.

[15] Michals K, Lopus M, Matalon R (1988). Phenylalanine metabolites as indicators of dietary compliance in children with phenylketonuria.. Biochem Med Metab Biol.

[16] McDonald JD, Bode VC, Dove WF, Shedlovsky A (1990). Pah^hph-5^: A mouse mutant deficient in phenylalanine hydroxylase.. Proc Natl Acad Sci USA.

[17] Shedlovsky A, McDonald JD, Symula D, Dove WF (1993). Mouse models of human phenylketonuria.. Genetics.

[18] Sarkissian CN, Boulais DM, McDonald JD, Scriver CR (2000). A heteroallelic mutant mouse model: A new orthologue for human hyperphenylalaninemia.. Mol Genet Metab.

[19] Buters JT, Sakai S, Richter T, Pineau T, Alexander DL (1999). Cytochrome P450 CYP1B1 determines susceptibility to 7, 12-dimethylbenz[a]anthracene-induced lymphomas.. Proc Natl Acad Sci U S A.

[20] Aldaz CM, Liao QY, LaBate M, Johnston DA (1996). Medroxyprogesterone acetate accelerates the development and increases the incidence of mouse mammary tumors induced by dimethylbenzanthracene.. Carcinogenesis.

[21] Keshava N, Mandava U, Kirma N, Tekmal RR (2001). Acceleration of mammary neoplasia in aromatase transgenic mice by 7,12-dimethylbenz[a]anthracene.. Cancer Lett.

[22] Fisher SM, Conti CJ, Locniskar M, Belury MA, Maldve RE (1992). The effect of dietary fat on the rapid development of mammary tumors induced by 7,12-dimethylbenz(a)anthracene in SENCAR Mice.. Cancer Res.

[23] Wurz GT, Read KC, Marchisano-Karpman C, Gregg JP, Beckett LA (2005). Ospemifene inhibits the growth of demethylbenzanthracene-induced mammary tumors in Sencar mice.. J Steroid Biochem Mol Biol.

[24] Huang M-T, Lou Y-R, Xie JG, Ma W, Lu Y-P (1998). Effect of dietary curcumin and dibenzoylmethane on formation of 7,12-demethylbenz[*a*]anthracene-induced mammary tumors and lymphomas/leukemias in Sencar mice.. Carcinogenesis.

[25] Qing W-G, Conti CJ, LeBate M, Johnston D, Slaga TJ (1997). Induction of mammary cancer and lymphoma by multiple, low oral doses of 7,12-dimethylbenz[*a*]anthracene in SENCAR mice.. Carcinogenesis.

[26] Liu J, Li J, Sidell N (2007). Modulation by phenylacetate of early estrogen-mediated events in MCF-7 breast cancer cells.. Cancer Chemother Pharmacol.

[27] Horlings H, Demant P (2005). Lung tumor location infiltration in mice are genetically determined.. Exp Lung Res.

[28] Hanigan MH, Winkler ML, Drinkwater NR (1990). Partial hepatectomy is a promoter of hepatocarcinogenesis in C57BL/6J male mice but not in C3H/HeJ male mice.. Carcinogenesis.

[29] Kaufman S (1999). A model of human phenylalanine metabolism in normal subjects and in phenylketonuric patients.. Proc Natl Acad Sci U S A.

[30] Kaur H, Halliwell B (1994). Aromatic hydroxylation of phenylalanine as an assay for hydroxyl radicals.. Analyt Biochemn.

[31] Fellman JH, Fujita TS, Roth ES (1972). Assay, properties and tissue distribution of *p*-hydroxyphenylpyruvate hydroxylase.. Biochim Biophys Acta.

[32] Linblad B, Lindstedt G, Lindstedt S, Rundgren M (1977). Purification and some proterties of human 4-hydroxyphenylpyruvate dioxygenase (I).. J Biol Chem.

[33] Tuchman M, Fisch RO, Ramnaraine ML, Krivit W (1985). Acidic metabolites of phenylalanine in plasma of phenylketonurics.. Biochem Med.

[34] Sidell N, Pasquali M, Malkapuram S, Barua AB, Wanichkul T (2003). In vitro and in vivo effects of easily administered, low-toxic retinoid and phenylacetate compounds on human neuroblastoma cells.. Br J Cancer.

[35] Doostdar H, Burke MD, Mayer RT (2000). Bioflavonoids: selective substrates and inhibitors for cytochrome P450 CYP1A and CYP1B1.. Toxicol.

